# Editorial: Aquaculture animal diseases: pathogens and control

**DOI:** 10.3389/fcimb.2023.1223046

**Published:** 2023-05-31

**Authors:** Rongrong Ma, Yibin Yang, Haipeng Cao, Pengfei Li

**Affiliations:** ^1^ School of Marine Sciences, Ningbo University, Ningbo, China; ^2^ Yangtze River Fisheries Research Institute, Chinese Academy of Fishery Sciences, Wuhan, China; ^3^ National Pathogen Collection Center for Aquatic Animals, Shanghai Engineering Research Center of Aquaculture, Shanghai Ocean University, Shanghai, China; ^4^ Guangxi Key Laboratory of Aquatic Biotechnology and Modern Ecological Aquaculture, Guangxi Academy of Marine Sciences, Guangxi Academy of Sciences, Nanning, China

**Keywords:** aquatic disease, diagnosis, pathogens, prevention and control, antibiotic evaluation

Aquaculture industry provides high quality animal protein in human diet. However, spread of diseases has been increasingly recorded, which constrained the aquaculture production. Timely diagnosis and discovery of pathogens and effective prevention and control of diseases are very important for healthy aquaculture.

It is our pleasure to present the Research Topics of *Aquaculture Animal Diseases: Pathogens and Control* to the readers. The present Research Topic is focused on Pathogens and Control of Aquaculture Animal Diseases and collects 16 articles, including three brief research report and thirteen original research articles from a total of 120 authors. The contributions cover research on diagnosis of pathogen, pathogenic mechanism, prevention and control methods of pathogens, and safety assessment of prevention and control measures.

*Carassius auratus* herpesvirus (CaHV) could induce fatal viral disease with high mortality of crucian carp (*Carassius auratus*), simple recombinase-aid amplification (RAA) assay coupled with lateral flow dipstick (LFD) were established, which could achieve sensitive diagnosis of tumor necrosis factor receptor (TNFR) of CaHV within 35 min at 40°C (Gui et al.). *Vibrio ponticus* is a vital pathogen with potential danger for aquaculture animals. And *V. ponticus* was isolated from diseased coral trout suffering liver necrosis with cell vacuolar degeneration. The identification and pathogenicity study of *V. ponticus* to the coral trout provide a reference for the control of pathogenic *V. ponticus* in the coral trout (Gai et al.). The bacterium *Elizabethkingia miricola* is a multispecies pathogen associated with meningitis-like disease were isolated from several amphibian species, including the bullfrog (Wei et al.). *Staphylococcus sciuri* was diagnosed in a sturgeon farm based on 16S rRNA gene phylogenetic analysis combined with biochemical identification, and some recommendations for treatment was provided (Zhang et al.).

*Citrobacter freundii* could cause the damage of the crayfish (*Procambarus Clarkii*) digestive organs by disrupting the intestinal microbiota to disturbed intestinal-liver axis homeostasis, which provide new insights into the pathogenic molecular mechanisms of *C. freundii* in the infection of crayfish (Li et al.). *Shewanella putrefaciens* was identified as a novel pathogen of the largemouth bass (*Micropterus salmoides*) and histopathological changes were observed in the intestine, head kidney, spleen, and liver of diseased fish (Jiang et al.). *Aeromonas salmonicida* is a typical cold water bacterial pathogen that causes furunculosis in many freshwater and marine fish species worldwide. Type II secretion system was found in the genome of *A. salmonicida*. And tatA, tatB and tatC regulate the virulence of *A. salmonicida* SRW-OG1 by affecting biofilm formation (Yi et al.).

In addition to bacteria, parasites also have been reported. *Pennella* sp. from the yellowfin tuna (*Thunnus albacares*) was separated, and morphological observation and molecular identification using the mitochondrial genome were carried out. This provide a fundamental basis for identifying parasites in yellowfin tuna and other fish, expand people’s understanding of parasites, and lay a foundation for the occurrence and prevention of parasites (Liu et al.).

In pathogen control, biological control and chemical drug control are studied. And its potential hazards are assessed. Bacteriophages, a class of viruses that lyse bacteria, can control drug-resistant bacteria. A novel *Vibrio parahaemolyticus* phage phiTY18 isolated from the coastal water of Xiamen was explored (Liu et al.). Dietary supplementation of *Bacillus amyloliquefaciens* can effectively improve the growth performance, digestive enzyme activity, immune responses, intestinal microbiota composition and disease resistance of yellow catfish (Xue et al.). The multidrug-resistant *Aeromonas hydrophila* was identified, and the mechanism of drug resistance was explored (Guo et al.). The occurrence of antibiotics and potential health risk of 300 cultured fish samples from 19 provinces in China were investigated. The high detection frequency and levels of antibiotics were found in samples (Tang et al.).

Overall, some new pathogens, virulence mechanisms, and prevention and control methods were explored. These results can provide important information for the prevention and control of aquaculture and epidemic disease prevention and control, help to develop effective disease control strategies, greatly improve the substitution rate of chemical drugs (antibiotics), effectively reduce the loss of aquaculture diseases, so as to guarantee the high quality development of aquaculture industry. We hope and expect the aquaculture research community will find this Research Topic of articles within pathogens and control topic informative and inspiring. As editors, we would like to thank the authors for their interesting contributions, as well as express our gratitude to all referees for their careful evaluation of the papers. Many thanks to all the colleagues who responded to this call, but whose interests could not be accommodated within the confines of this Research Topic. Finally, we extend our sincere appreciation to Frontiers in Cellular and Infection Microbiology for supporting this exciting Research Topic.

## Author contributions

RM and YY prepared the draft editorial. HC and PL revised the manuscript. All authors contributed to the article and approved the submitted version.

